# A rapid point-of-care assay accurately measures vitamin D

**DOI:** 10.1007/s40618-021-01575-8

**Published:** 2021-04-22

**Authors:** K. Albrecht, J. Lotz, L. Frommer, K. J. Lackner, G. J. Kahaly

**Affiliations:** 1 Department of Medicine I, Johannes Gutenberg University (JGU) Medical Center, Mainz, Germany; 2 Institute for Clinical Chemistry and Laboratory Medicine, Johannes Gutenberg University (JGU) Medical Center, Mainz, Germany

**Keywords:** Vitamin D serum levels, Point-of-care, Rapid assay, Immunofluorescence assay, Chemiluminescence assay

## Abstract

**Purpose:**

Vitamin D (VitD) is a pleiotropic hormone with effects on a multitude of systems and metabolic pathways. Consequently, the relevance of a sufficiently high VitD serum level becomes self-evident.

**Methods:**

A rapid immunofluorescence assay designed for the point-of-care measurement of serum VitD_3_ solely was tested. Inter- and intra-assay validation, double testing and result comparison with a standardized laboratory method were performed.

**Results:**

An overall linear correlation of *r* = 0.89 (Pearson, 95% CI 0.88–0.92, *p* < 0.01) between the point of care and the conventional reference assay was registered. Accuracy and precision were of special interest at cut-points (10 ng/ml [mean deviation 1.7 ng/ml, SD 1.98 ng/ml, SE 0.16 ng/ml], 12 ng/ml [MD 0.41, SD 1.89, SE 0.19] and 30 ng/ml [MD − 1.11, SD 3.89, SE 0.35]). Only a slight deviation was detected between the two assays when using fresh (*r* = 0.91, 95% CI 0.86–0.94, *p* < 0.01) and frozen serum samples (*r* = 0.86, 0.82–0.89, *p* < 0.01). Results remained steady when samples were frozen several times. Inter- and intra-assay validation according to the CLSI protocol as well as multiuser testing showed stable results.

**Conclusion:**

This novel, innovative, and controlled study indicates that the evaluated rapid point of care VitD assay is reliable, accurate, and suited for clinical practice.

## Introduction

Vitamin D (VitD) is important for healthy living, and its pleiotropic effects go far beyond the mineral metabolism [1–4]. VitD measurement is especially recommended in patients with bone disorders and/or pathologies in calcium homeostasis. VitD is an active steroid hormone, either synthesized from cholesterol or taken in dietary in the form of Cholecalciferol (D_3_) or the less potent Ergocalciferol (D_2_). However, with the main dietary sources for D_3_ being fish and for D_2_ only eggs, milk, and mushrooms, one would not be able to reach sufficient uptake. Thus, the majority of circulating D vitamins originate from bodily production. After hepatic dehydrogenation as well as ultraviolet radiation B (280–315 nm) and heat dependent processing in the skin, the precursor D_3_ is produced [5]. Subsequently, D_3_ is transported back to the liver and converted to 25(OH)D_3_ by several cytochrome P450 mixed-function oxidases (CYP), such as CYP27A1, CYP3A4, and especially CYP2R1 [6, 7]. 25(OH)D_3_ is the storage form of VitD, and consequently due to its half-life of 2–3 weeks [8], the best marker to monitor VitD status. 1.25(OH)_2_D_3_ may impact pathogenesis and prognosis of chronic, malignant, and endocrine disease [9–11].

This study was designed to validate a point-of-care diagnostic device for rapid 25(OH)D_3_ testing. If established, it can be used by primary care practitioners and general physicians, as well as pharmacies and specialized practices without laboratory connection to optimize 25(OH)D_3_ supplementation.

## Materials and methods

### Samples

Serum samples (*n* = 324) were collected from unselected, consecutively followed subjects with diagnosed endocrine and non-glandular diseases at the academic tertiary referral center for endocrine diseases of the Johannes Gutenberg University (JGU) Medical Center, Mainz, Germany.

Ethical approval was obtained by the local Ethical Committee of the Medical Chamber of the state Rhineland-Palatinate, Germany, and written informed consent was obtained from all subjects. Including validation testing, a total of 433 test runs were performed. Serum was taken from subjects independent of season and either stored or tested immediately after collection. Stored samples were frozen at − 20 °C (− 4°F). Fresh and frozen samples were each statistically analyzed separately as well as together.

### Index assay—Sofia device

The Sofia Quantitative Vitamin D FIA is a point-of-care (POC) immunofluorescence-based lateral flow assay (Quidel Inc., San Diego, USA). It is designed for in vitro quantitative determination of total 25(OH)D_3_ using serum samples. Two steps are of immense importance, first serum preparation and secondly reagent application to the test strip. Quantitative testing ability ranges from 10 to 100 ng/ml. If the serum 25(OH)D_3_ lies below or above aforementioned limits, test results will correspondingly be shown as: “ < 10 ng/ml” or “ > 100 ng/ml”. Complying with CDC recommendation in manufacturer validation, the index assay was correlated with the LC–MS/MS reference method (*r* = 0.91 [95% CI 0.87–0.94]) and thus met VDSP performance criteria [12]. The Quidel Sofia FIA is able to run a high number of point of care assays, which were previously tested. To the extent of our knowledge, there is currently no clinical practice study concerning the total 25(OH)D_3_ POC.

#### Index assay sample processing

To perform the test, a blood sample is collected from the patient and immediately centrifuged at 1300×*g* for 10 min. Frozen or refrigerated samples should be well tempered and well mixed by inverting at least ten times or vortexing. A calibrated micropipette is used to add 100 μl of the patient serum sample to the reagent vial, which is then inverted two times for the contents to mix. Subsequently, 5 min of incubation follow during which 25(OH)D_3_ molecules are released from VitD-binding proteins (DBP). During incubation, a test mode is selected and user and patient ID are put in either manually or by scanning a barcode. On pressing “start test”, a drawer will automatically open. Immediately after five minutes, a micropipette is used to withdraw 120 μl of treated solution from the reagent vial and dispensed into the sample well on the test cassette which contains sheep monoclonal anti-25(OH)D_3_. The foil pouch containing the test cassette was opened 20 s before immediate use, avoiding unnecessary environmental exposition as recommended. The test strip contains chemical environments that produce a fluorescent signal indicative for the concentration of total 25(OH)D_3_ in the patient sample. The fluorescent signal is invisible and must be interpreted by the corresponding device. The test cassette is inserted into the drawer, which is then manually closed. Five minutes of test development start, during which the sample migrates the test strip. Sofia scans the test strip and analyzes the fluorescent signal using method-specific algorithms. Sofia then displays the quantitative result of total 25(OH)D_3_ on the screen and optionally prints the result with an integrated printer [13].

### Validation of the point of care index assay

To validate and describe the performance data of the device, we conducted several test series with different approaches. The following parameters were determined: random error, constant error, and proportional error. The total validation was divided into two parts [14]: first, the familiarization with the POC device and, second, the experimental evaluation, which itself was divided into a preliminary and final part. The preliminary part calculated the random error, interferences, and recovery. The implementation of the method comparison was part of the final evaluation experiment. The determination of the inter-assay imprecision was replaced by the calculation of the total CV. For determination of random error (intra-assay variation), POC units (*n* = 20) were prepared and measured directly one after another within 1 day. In addition, inter- and intra-assay testing was performed according to the CLSI protocol over the course of 5 days, in the morning and the evening, respectively, adding up to 20 (in total *n* = 40) test runs each. The two chosen VitD serum concentrations reflect 25(OH)D_3_-levels of interest, with one being sufficient (30–80 ng/ml) and one being insufficient (< 30 ng/ml) [15]. Testing for linearity, a recovery experiment was conducted. Two samples with high serum 25(OH)D_3_ were chosen on behalf of the reference test result. Eleven dilutions were prepared each (100, 90, 80, 70, 60, 50, 40, 30, 20, 10, and 0%), using patient serum sample and Dulbecco’s Phosphate Buffered Saline (PBS) as diluent. The diluted solution was then tested using the index assay, the results were compared to calculated target values, and the difference between both values was noted. The stability of VitD in serum was verified by testing two samples, frozen and thawed every day, for up to seven cycles over the course of 9 days. Regarding that POC devices are often used by several employees, inter-operator reliability was investigated by five users testing three samples. For method comparison, the POC assay was compared with an established chemiluminescence assay of an automated analyzer. The slope and intercept of the linear regression equation describe the constant and proportional error of the methods. In addition, the coefficient of correlation was calculated.

### Reference conventional assay—Abbott Alinity i

For reference and control, each serum sample was also measured at the JGU central laboratory using an established automated immunoassay. The Alinity i 25-OH Vitamin D Reagent Kit (Abbott, USA) is a delayed one-step immunoassay for quantitative in vitro determination of 25(OH)D_3_. It is based on the technique of chemiluminescence microparticle immunoassays (CMIA). This assay is standardized against NIST SRM 2972 (National Institute of Standards & Technology Standard Reference Material 2972) and Clinical and Laboratory Standards Institute (CLSI). For validation, results were compared to isotope dilution liquid chromatography with tandem mass spectrometry (LC–MS/MS) [16]. For this study, only serum samples were used. Quantitative testing ability ranges from 3.5 to 154.2 ng/ml. If the serum 25(OH)D_3_ lies below or above aforementioned limits, test results will correspondingly be shown as: “ < 3.5 ng/ml” or “ > 154.2 ng/ml”.

#### Reference assay work flow

Sample, paramagnetic with anti-VitD-coated microparticles, and assay diluent are mixed and incubated. The 25(OH)D_3_ in the sample is separated from the VitD-binding protein (DBP) and binds to the anti-VitD-coated microparticles. The VitD-acridinium-labeled conjugate is added and a reaction mixture is prepared. The reaction mixture is incubated. After a washing cycle, pre-trigger and trigger solutions are added. The resulting chemiluminescence reaction is expressed in relative light units (RLE) and is measured. The amount of 25-OH VitD in the sample is proportionally related to the RLE measured by the optical system. It takes 1.5 h from sample input to receipt of the result [16].

### Statistical analysis

Data analysis was performed using Microsoft Excel 16.39 (Microsoft Corporation, Redmond, Washington, USA) and MedCalc® Statistical Software version 19.6 (MedCalc Software Ltd, Ostend, Belgium; https://www.medcalc.org; 2020). Pearson and Lin correlation were implemented, as well as Passing Bablok regression [17, 18], which is used to detect correlations as well as constant and proportional bias between the index point of care and reference assay results. The model is suited for method comparison due to its immunity towards outliners. Results are also presented as Bland–Altman plot to show the difference between index and reference results plotted against their mean. This allows to find a correlation between a measurement error and the estimated true value [19]. The significance level was 5% (*α* = 0.05).

## Results

**Fig. 1:**
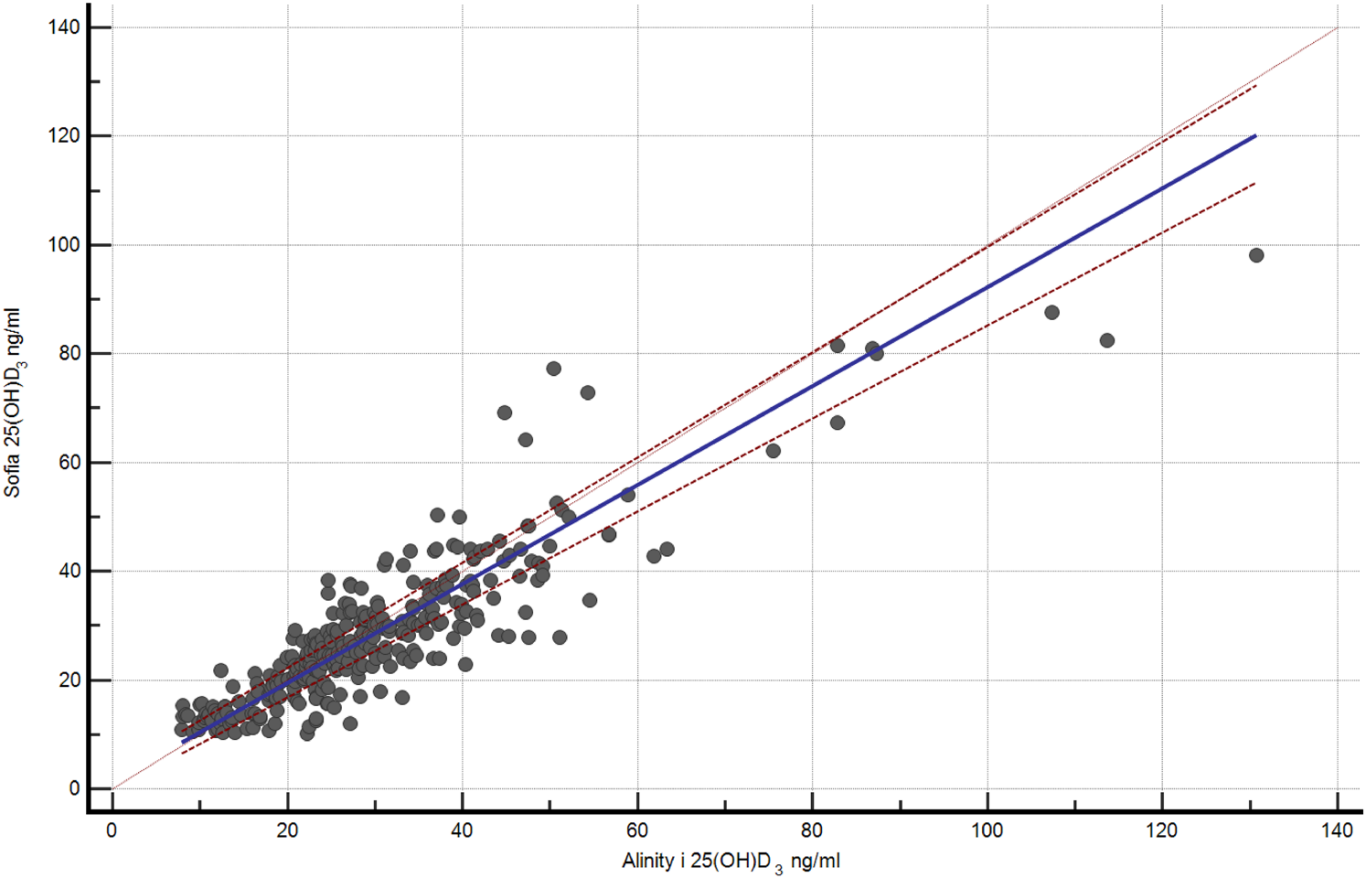
Passing Bablok regression for all samples. *N* = 296 samples, Pearson correlation *r* = 0.89 (95% CI 0.88–0.92, *p* < 0.01), y = 1.312 + 0.910x. Intercept A = 1.31 (95% CI − 0.27 to 2.9. Slope B = 0.91 (95% CI 0.86–0.97)

**Fig. 2:**
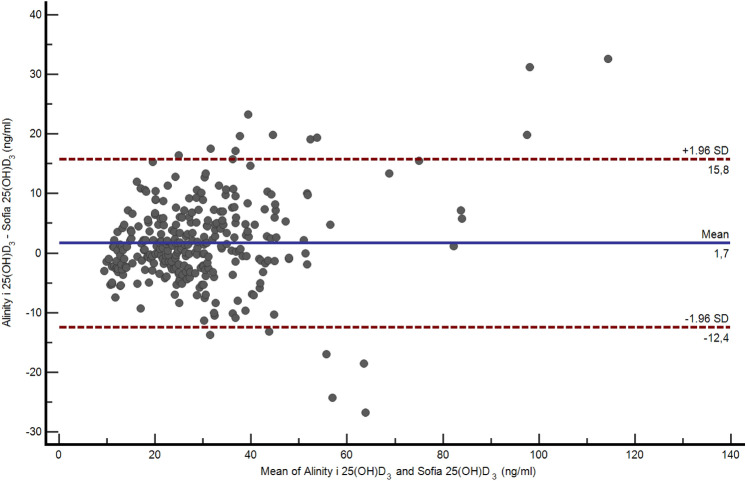
Bland–Altman plot for all samples. It shows the difference in results of both assays plotted against their means. The values scatter coincidentally, without systematic deviation

**Fig. 3:**
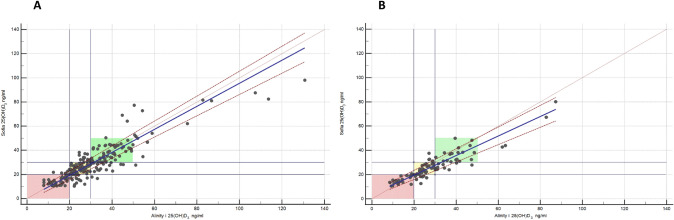
**a**, **b** Comparison of fresh versus frozen samples. **a** Passing Bablok regression for frozen samples (*n* = 208), *r* = 0.89 (95% CI 0.85–0.91), *p* < 0.01, y = 0.120 + 0.954 x, *r*_*c*_ = 0.87 (95% CI 0.84–0.89). **b** Passing Bablok regression for fresh samples (*n* = 88), *r* = 0.92 (95% CI 0.87–0.94), *p* < 0.01, y = 3.506 + 0.805 x, *r*_*c*_ = 0.89 (95% CI 0.87–0.91). Green: sufficient VitD serum levels (30–80 ng/ml), yellow: insufficient levels (20–29 ng/ml), red: deficient serum levels (< 19 ng/ml). Sofia (Quidel) = Index assay; Alinity i (Abbott) = reference assay; frozen samples were stored at − 20 °C (− 4°F), fresh samples were tested immediately after collection and sample preparation

**Fig. 4:**
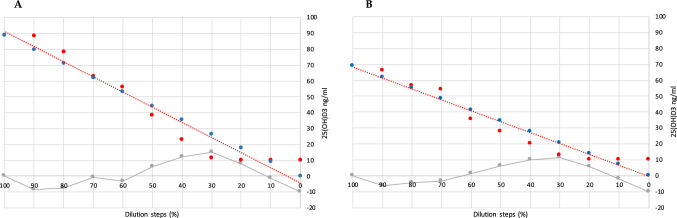
**a**, **b** Test for linearity. Two serum samples were diluted eleven times each (100, 90, 80, 70, 60, 50, 40, 30, 20, 10 and 0%) **a** Dilution of a serum sample at 89 ng/ml VitD. Deviation ranges from − 8 to 15 ng/ml maximum, mean = 0.9 ng/ml (SD = 8.4 ng/ml). **b** Serum level of 66 ng/ml VitD. Deviation ranges from − 6 to 7 ng/ml maximum, mean = 0.6 ng/ml (SD = 6 ng/ml). Blue: Target value, red: measured result, grey: difference between expected and measured value (ng/ml)

**Fig. 5:**
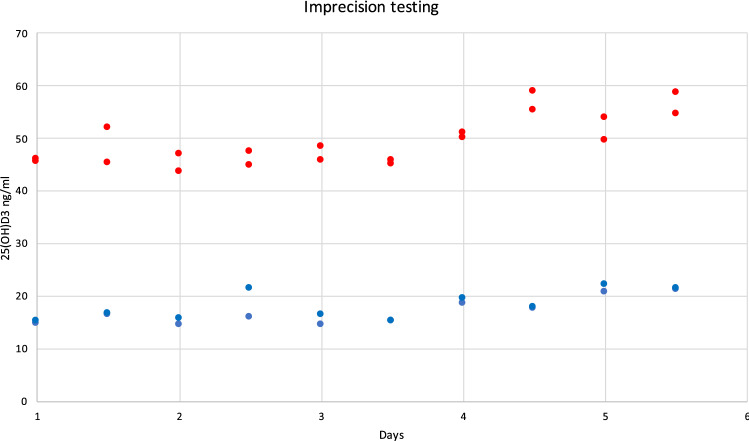
Determination of imprecision. Testing for total CV according to the CLSI protocol. Two serum samples were tested four times a day, twice in the morning and twice in the evening over the course of 5 days. Total CV was 15.1 and 9% for the samples with low and high VitD levels, respectively. Blue and red along one trendline represent the lower (blue) and higher (red) test result

**Fig. 6:**
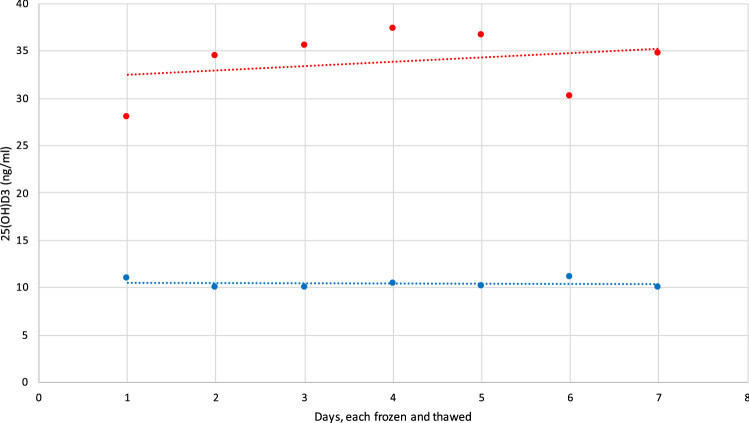
Inter-assay and stress testing. N = two samples, frozen and thawed every day over the course of 7 days. SD ranged from 0.5 ng/ml (low serum VitD, blue) to 3.5 ng/ml (high serum VitD, red), CV from 5 (low VitD) to 10% (high VitD)

A. **Demographic and serological data**
  A total of 324 serum samples from unselected, consecutively followed outpatients with endocrine and non-endocrine diseases, were collected. In detail, patients with various thyroid diseases, type 1 and 2 diabetes, metabolic syndrome, monoglandular and polyglandular autoimmunity were included. Individual medication was considered during analysis. Of these, 296 samples were included in this comparison study (229 females and 67 males, 156 smokers), with an average age of 48.1 years (range 6.8–84.0 years). Twenty-eight samples outside of the measuring range of the index assay (10–100 ng/ml) were documented and then excluded due to falsification of correlation results. The falsification of the results is caused by the limitations of the measuring range. Values far above or below this range will result in large absolute deviations if viewed out of context, although the index assay can correctly indicate that the value is outside its range. Of the 28 excluded samples, one sample only was above 100 ng/ml, while 27 samples were below 10 ng/ml. 18 of these samples were also measured < 10 ng/ml by the Abbott Alinity i and nine were measured > 10 ng/ml**.** Furthermore, serum samples were divided into fresh (*N* = 88) and frozen samples (*N* = 208). A total of 45 patients reported VitD substitution, of whom only one showed 25(OH)D_3_ serum levels above 100 ng/ml.
B. **Overall results with both devices**
  The arithmetical mean for the index assay was 28.5 ng/ml (SD [standard deviation] 14.17 ng/ml; SE [standard error] 0.82 ng/ml; CV [coefficient of variation] 50%), while the median was 24.6 ng/ml. In comparison, the arithmetical mean for the reference assay and the median were 30.2 ng/ml (SD 15.96 ng/ml; SE 0.93 ng/ml; CV 53%) and 25.7 ng/ml, respectively (*p* < 0.01). The correlation factor was 0.89. Due to the design of the study, it was verified using Lin’s Concordance Correlation Coefficient (Lin_CCC_; r_c_), which showed minimal deviation (*r*_*c*_ = 0.88; 95% CI 0.85–0.9) when compared to the Pearson’s correlation coefficient [20]. Accuracy and precision were calculated at 10 ng/ml (95% CI ± 1.21, mean deviation 1.7 ng/ml, SD 1.98 ng/ml, SE 0.16 ng/ml), 12 ng/ml (95% CI ± 1.1, MD 0.41, SD 1.89, SE 0.19), and 30 ng/ml (MD − 1.11, SD 3.89, SE 0.35). The Passing Bablok regression and the Bland–Altman plot analysis are shown in Figs. 1 and 2. Aiming to see whether or not frozen serum samples would produce a comparably strong test power, fresh (Index assay median = 24.8 ng/ml [SD 11.7 ng/ml], reference assay median = 26.1 ng/ml [SD 14.4 ng/ml]) and frozen samples (Index assay median = 27 ng/ml [SD 15 ng/ml], reference assay median = 27.3 ng/ml [SD 16.6 ng/ml]) were analyzed separately. The Passing Bablok regressions are shown in Fig. 3.
C. **Precision data of the Sofia point of care assay**
  The linearity was assessed using two dilution series (Fig. 4). Deviation ranges from − 6.3 to 11.5 ng/ml. Sofia test results for steps 20% to 0% were always displayed as:” < 10 ng/ml”. According to the CLSI protocol, two samples representing the region of interest at a low (mean = 17.58 ng/ml, SD = 2.61 ng/ml, CV 14.86, interassay CV 5.13) and a high serum level (mean = 49.5 ng/ml, SD = 4.72 ng/ml, CV = 9.53, interassay CV 7.34) were each measured two times a day (at 8 am and at 4 p.m.) in double determination. To test for imprecision, a total coefficient of variation (CV) was calculated and found at 15.1% for the sample with low VitD level and 9% for the sample with high VitD level (Fig. 5). Multi-operator validation was performed by five operators testing three samples at deficient, insufficient, and sufficient (13, 27 and 33 ng/ml) VitD serum levels up to two times. Standard deviation (SD [ng/ml]) ranged from 0.9 to 2.9; with a CV of 7–8%. Two samples were frozen and thawed every day over the course of 7 days, stress testing the stability of VitD in serum samples and verifying inter-assay variability (Fig. 6).

## Discussion

This novel and innovative controlled study demonstrates the accuracy of an easy to handle point of care device for the rapid and quantitative measurement of 25(OH)D_3_ in the serum of a large number of unselected and consecutively followed outpatients with various endocrine and non-glandular diseases. Compared to a conventional methodology in the university central lab, this small device offered acceptable and reliable serum VitD values with an overall correlation of approximately 90% between both assays. Although requests of the American/European Chemistry Societies and/or Laboratory Medicine for correlations around 98–99% prevail, the same standards cannot be applied for a point of care device. Hence, a second test with a standard method might be indicated if 25(OH)D values < 30 ng/ml are found, to properly address VitD supplementations according to national and international guidelines.

The rationale for this study was to compare the reliability and acceptable accuracy of this rapid, convenient, and simple methodology in daily routine. In the light of the above, we found a device that offers a very good comparability to an established method. The measuring range covers the clinically relevant spectrum from absolute deficiency to the toxic edge sufficiently. The results are stable over time and the device is easy to work with. Despite slightly lower concentration results with the Sofia device, the subgroup categorization between deficiency, insufficiency, and sufficiency is satisfactory. Once confirmed, point of care measurement of serum VitD could be performed reliably, rapidly, and easily in each clinical practice and pharmacy worldwide.

In detail, the coefficients of correlation were similar in fresh and frozen samples (*r* = 0.92 and *r* = 0.89, respectively), with a slightly smaller deviation when testing fresh samples. Results were less accurate for VitD serum levels above 100 ng/ml. Only one patient achieved correspondingly high values on VitD substitution, which is why currently, we cannot exclude the possibility that this impacts the assay in terms of specificity. While the standard reference method gave results in the toxic range, the point of care assay still measured below toxicity. Therefore, for concentrations above 80 ng/ml and to be on the safe side, we recommend an additional test with a standard method. The linearity check (dilution with buffer) showed a slight overestimation at dilutions with 10–20% dilution medium in the test preparation, and an underestimation at dilutions greater than 1:2. This could possibly be attributed to a matrix effect due to the higher non-protein content, evoking changed flow properties. The imprecision test was performed on frozen samples. Both sera were frozen once, thawed, and then stored in the refrigerator for 5 days. During these 5 days, the results increased slightly and a continuous drift could be observed. Whether this is due to the storage form and a possibly increasing instability of the VitD-DBP complex cannot be determined with certainty. Samples delivered stable results when frozen several times with a coefficient of variation of less than 10%; identical applies to multiple users testing the same serum sample. Finally, stress testing delivered very satisfying results. Overall, this indicates that the influence on the assay performance through handling is not critical. This is a very important aspect when operating a point of care analyzer.

The individual VitD status is influenced by diet, sun exposure, skin pigmentation, age, and BMI. Genetic factors also play an important role [9]. Several studies analyzed serum levels of 25(OH)D_3_ for a wide range of countries, showing that hypovitaminosis D is a worldwide issue of concern. Especially, pregnant women, newborn children, and elderly people are at high risk of insufficiency and its consequences [21]. As hypovitaminosis, hypervitaminosis D carries risks as well by 25(OH)D_3_ acting toxic above serum levels of 150 ng/ml [22]. Respectively, the American Endocrine Society advises to screen patients at risk for deficiency. 25(OH)D_3_ levels below 30 ng/ml are declared insufficient, while levels below 20 ng/ml rate as deficient [15]. Although the optimal serum level could not yet be determined, the American Geriatrics Society recommends minimum serum levels of 30 ng/ml as well [23]. While the importance of a sufficiently high VitD level is evident, the establishment of official guidelines and benchmarks has proven difficult, and still does so. This is due to the vast amount of different assays available within the field and lack of standardization resulting in wide intra-assay variation and inter-assay variability. Against the backdrop of this inconsistency, the interpretation of study results in form of systematic reviews is prevented. To achieve uniformly usable results and establish globally valid guidelines, the Vitamin D Standardization Program (VDSP) and the Vitamin D External Quality Assurance Scheme (DEQAS) were founded. The VDSP was created in 2010 by the National Institutes of Health (NIH), the Centers for Disease Control and Prevention (CDC) and others. The goal was and is to standardize new assays using the reference measurement procedures (RMPs) operated by Ghent University, National Institute for Standards and Technology (NIST) and the CDC. Both RMPs operate using isotope dilution liquid chromatography with tandem mass spectrometry (LC–MS/MS) and are defined as the gold standards regarding determination and measurement of the true 25(OH)D serum level [12, 24]. The DEQAS operating by CDC standardization has been monitoring laboratories assaying 25(OH)D quarterly over the last 30 years. Another factor that complicates the measurement of reliable values is the interference of variables with the assays. For example, the supplementation of ergocalciferol (D_2_) or varying affinity and release from vitamin D-binding protein (DBP) [25, 26]. Complying with VDSP recommendations, the introduced index point of care assay met CDC criteria.

In conclusion, the novel and encouraging data indicate that the evaluated point of care assay for the rapid measurement of serum VitD is reliable, accurate, and suited for clinical practice.
